# Predictive factors of the duration of sick leave due to mental disorders

**DOI:** 10.1186/s13033-019-0279-6

**Published:** 2019-03-30

**Authors:** Sawako Sakakibara, Mitsuhiro Sado, Akira Ninomiya, Mayuko Arai, Satoko Takahashi, Chika Ishihara, Yuki Miura, Hajime Tabuchi, Joichiro Shirahase, Masaru Mimura

**Affiliations:** 1 Center for Counseling and Disability Services, Tohoku University, Sendai, Japan; 2 Department of Neuropsychiatry, Keio University School of Medicine, Shinanomachi 35, Shinjuku-ku, Tokyo, 160-8582 Japan; 3 Center for Stress Research, Keio University, Tokyo, Japan

**Keywords:** Return to work, Mental disorders, Sick leave, Employee, Occupational mental health

## Abstract

**Background:**

This study aimed to examine potential predictors of duration of sick leave due to mental disorders in Japan.

**Methods:**

A total of 207 employees at a manufacturing company in Japan with a past history of sick leave due to mental disorders participated in this study. Mental disorders were defined as those listed in the Diagnostic and Statistical Manual of Mental Disorders, Fourth Edition (DSM-IV). All of the participants used the mental health program that the company provided. The predictive power of the variables was tested using a Cox proportional hazard analysis. The hazard ratios in the final model were used to identify the predictor variables of the duration of sick leave. We included socio-demographic (age, sex, tenure), clinical (diagnosis and number of previous sick leave), and work-related factors (employment rank) as possible predictors. Data on these variables were obtained through the psychiatrists and psychologists in the company’s mental health program.

**Results:**

The results of the univariate analyses showed that the number of previous sick leave episodes, diagnosis and employee rank were significant predictors of the duration of sick leave due to mental disorders. A multivariate analysis indicated that age, number of previous sick leave and employee rank were statistically significant predictors of return to work.

**Conclusions:**

Diagnosis, number of previous sick leave episodes, and employee rank are predictors of the duration of sick leave due to mental disorders. This study’s findings have implications in the development of effective interventions to prevent protracted sick leave.

## Background

Mental disorders are highly prevalent in the general population, and cause considerable burden to society [1–3]. Previous studies in Japan indicated that the total costs of mental disorders are enormous. Total cost of schizophrenia, depression, and anxiety disorder in Japan in 2008 was Japanese Yen (JPY) 2.77 trillion (United States Dollars (USD) 23.8 billion), JPY 3.09 trillion (USD 26.5 billion), and JPY 2.39 trillion (USD 20.5 billion), respectively [4, 5]. When it comes to the workplace, it is well known that mental disorders contribute to sick leave of employees in Western countries [1, 6–8] and also in Japan [9]. Mental disorder is one of the main causes of absenteeism in employees [10, 11]. Despite the enormous negative effects of sick leave due to mental health problems, our knowledge about prognostic factors for the duration of sick leave is still very limited [12, 13].

The duration of sick leave due to mental disorders, such as adjustment disorder, depression, anxiety disorder, and schizophrenia, is longer than that due to physical disorders [14]. The costs of sick leave caused by mental disorders are extremely high not only for the individual but also for their workplace and society [3, 15, 16]. Therefore, delays in returning to work result in high compensation costs. Nevertheless, studies on return-to-work rehabilitation programs for workers with mental disorders are very limited [12, 13, 17]. A better understanding of the prognostic factors for longer sick leave would be helpful to develop effective prevention and intervention strategies for reducing the costs and personal distress associated with longer-term sick leave.

A systematic review article by Cornelius et al. [12] revealed that older age (> 50 years) was strongly associated with longer duration of sick leave until returning to work. However, it is not clear whether this finding could be applicable to non-Western countries like Japan, where the healthcare system and employment contracts regarding sick leave differ from those in Western countries. For example, it is fairly common for the employees of Japanese large companies to have the right to take sick leave over 3 years. To the authors’ knowledge, there have been few studies that have investigated this issue in Japan. The only study in Japan relevant to this context is the one conducted by Sado et al. [18]. However, the focus of the Sado et al’s study lies on the risk factors for ‘repeated sick leave’ after return to work. With respect to the risk factors for ‘duration of sick leave’ in this study, there have been no studies to the authors’ knowledge. Therefore, we decided to evaluate the variables, relevant to socio-demographic, clinical, and work related, that predict duration of sick leave due to mental disorders in Japan.

## Objective

The objective of this study is to examine the variables, relevant to socio-demographic, clinical, and work related, that predict duration of sick leave due to mental disorders in Japan.

## Methods

The method adopted for this study follows that described in the previous study by Sado et al. [18], the same data set was utilized. However, the variables included in the analysis of this study differs from the earlier study. The dependent variables in Sado et al. [18]. was the number of survival days (the duration between the return to work and the repeated sick leave). On the other hand, the dependent variable of this study is the number of sick leave days (the duration between starting sick leave and returning to work).

### Design

This was a retrospective cohort study. This study was approved by the Clinical Research Ethics Committee at Keio University School of Medicine (reference: 2013-485).

### Participants

This study was carried out in a company with approximately 10,000 employees in the Tokyo metropolitan area. The company is one of the most well-known manufacturing companies in Japan, mainly developing home electrical products.

Data was derived from 207 workers of the company. Participants started their sickness absence from work between April 1, 2009 and March 31, 2012. All employees were included in this study, who took sick leave longer than successive 20 days due to their mental disorders during that period. The reason for the cutoff point of 20 days was that in the company’s regulations, employees who took over successive 20 days sick leave must have undergone an assessment by an occupational psychiatrist to determine whether they were mentally fit to return to work in the rehabilitation program. The program also includes reinstatement support by occupational psychiatrists and psychologists. All participants in this study utilized the program.

Clinical diagnoses in this study were made by a senior occupational psychiatrist according to the criteria of Diagnostic and Statistical Manual of Mental Disorders, fourth edition (DSM-IV). He judged on the basis of the information from psychiatrists and psychologists in the program who had sessions with the participants, their co-workers and personnel in the company’s human resource division. The diagnosis affecting the most recent sick leave was coded as primary diagnosis. When participants took sick leave due to both physical and mental disorders, they were included in the analyses only if the main reason for sick leave was judged to be mental disorders. In the case of multiple psychiatric diagnoses, the disorder playing the strongest role in the sickness absence was coded as the diagnosis and used for analyses in this study.

### The process of return to work after sick leave in the company

Soon after a worker submits a medical certificate for returning to work to the human resources division, they are referred to the occupational mental health support team in the company. They then participate in the rehabilitation program provided by the team, using cognitive behavioral therapy components such as behavioral activation and activity scheduling. In the program, after four sessions of the intervention, workers are evaluated by the team psychiatrist in terms of their fitness to work. After this process is successful, workers return to work with some restrictions on engagement, such as no overtime duties for a period of time. The restrictions on engagement are expected to be gradually reduced over 6 months until they are lifted completely.

### Variables in the study

#### Dependent variable

The dependent variable was the number of sick days off due to mental disorders. We counted the duration between starting to take sick leave due to mental disorders and return-to-work or March 31, 2012 if they didn’t return to work at that time.

#### Explanatory variables

Although various factors are likely to predict return to work [1, 19], our choice of explanatory variables in this study was based on the difficulty in obtaining information of various types and data on many variables through time-consuming processes such as structured interviews with employees returning to work during their usual rehabilitation program activities. Therefore, we judged it more appropriate in these circumstances to use readily accessible variables that are likely to prolong absence from work. The following were chosen as explanatory variables in this study.

1. Socio-demographic factors:

Age of participant when sick leave started (20–29 years, 30–39 years, 40–49 years, and 50 years and older), sex, age at start of employment (under 25, 25–29, 30 or over) and job tenure (under 5, 5–9, 10–19, over 19 years).

2. Clinical factors:

Diagnosis (major depressive disorder, adjustment disorder, bipolar disorder, anxiety disorder, schizophrenia, and other disorders), and number of previous sick leave (none, one, and two and more).

3. Work-related factors:

Employee rank (assistant staff, middle staff, senior staff, and managers); employee rank in the company is strongly related to the job role; typical job roles for each employee rank are described below.

Assistant staff: expected to support other staff in working efficiently; typically, assistant staff have graduated from high school or junior college.

Middle staff: expected to conduct tasks assigned by senior staff and to usually follow instructions from their team leaders to conduct their work; typically, each worker stays in this position for 5–10 years after graduating from university or graduate school.

Senior staff: expected to work as leaders of their team, which typically consists of middle and assistant staff; their mission is to manage their team to carry out the tasks assigned by their manager; they are also expected to communicate efficiently with other senior staff working as team leaders and managers in order to conduct their projects successfully; typically, senior staff members are in their mid-30 s to 40 s.

Managers: expected to operate their projects by managing several teams; their main roles are to provide team leaders with objectives and oversee the development of projects through discussions with team leaders; typically, they are over 40 years of age.

### Analysis

Firstly, we examined the characteristics of the participants. Subsequently, univariate analysis was conducted. The dependent variable (number of sick days off work due to mental disorders) was censored when participants’ sick leave continued until March 31, 2012, or if they retired or transferred out of the Tokyo metropolitan branch by this date. Each explanatory variable was assessed using the log-rank test. Next, Cox proportional hazard analysis was conducted to identify relevant predictors of the duration of sick leave using a stepwise forward selection method. All analyses were conducted with SPSS for Windows, version 19, the license for which was provided by Keio University School of Medicine.

In this study, we utilized secondary data that had been already obtained during the usual process of return to work in the company. As mentioned above, this maximizes practical value; that is, the potential predictors are easily available to occupational psychiatrists in this and other companies in Japan. In addition, we used only anonymous data. We conducted this study with opt out consent method.

## Results

### Characteristics of the participants

1. Socio-demographic factors

The mean age of start of sick leave in this study was 38.3 (*SD* = 7.39) years. Mean age at start of employment and job tenure were 25.6 (*SD *= 3.81) and 14.9 (*SD* = 8.13) years, respectively.

2. Clinical factors

While 68.1% (*n* = 141) of participants had never experienced an episode of sick leave before, 17.9% (*n* = 37) had experienced one previous sick leave episode due to mental disorders, and 14.0% (*n* = 29) had experienced two or more.

A total of 50.2% (*n* = 104) of participants were diagnosed with major depressive disorder, 27.5% (*n* = 57) with adjustment disorder, 6.3% (*n* = 13) with bipolar disorder, 3.4% (*n* = 7) with anxiety disorder, and 3.4% (*n* = 7) with schizophrenia. Other diagnoses (9.2%, *n* = 19) included personality disorders, substance related disorders, pervasive development disorders, disruptive behavior disorder, and unspecific mental disorders.

### Work-related factors

While 17.4% (*n* = 36) of participants were managers, 48.8% (*n* = 101), 30.0% (*n* = 62), and 3.9% (*n *= 8) were senior staff, middle staff, and assistant staff, respectively.

#### Duration of sick leave

The mean number of sick days off work due to mental disorders was 235.8 (*SD* = 228.4) days.

The details of the participant characteristics are shown in Table 1.

**Table 1:** Characteristics of the participants

Variables	n	%	Mean	SD
Socio-demographic				
Age at start of sick leave (years)			38.3	7.39
20–29	26	12.6		
30–39	96	46.4		
40–49	72	34.8		
≧ 50	13	6.3		
Sex				
Male	168	81.2		
Female	39	18.8		
Age at employment (years)			25.6	3.81
< 25	93	44.9		
25–29	86	41.5		
≧ 30	28	13.5		
Tenure (years)			14.9	8.13
< 5	22	10.6		
5–9	32	15.5		
10–19	87	42.0		
≧ 20	66	31.9		
Clinical				
Number of previous sick leave				
0	141	68.1		
1	37	17.9		
2≦	29	14.0		
Diagnosis				
Major depressive disorder	104	50.2		
Adjustment disorder	57	27.5		
Bipolar disorder	13	6.3		
Anxiety disorder	7	3.4		
Schizophrenia	7	3.4		
Others	19	9.2		
Duration of sickness absence (days)			235.8	228.4
Work related				
Employee rank				
Manager	36	17.4		
Senior staff	101	48.8		
Normal staff	62	30.0		
Assistant staff	8	3.9		

*SD* standard deviation

#### Univariate analysis

Univariate analysis was conducted. Each explanatory variable was assessed using the log-rank test. The number of days of previous sick leave, diagnosis, and employee rank were significant. With respect to diagnosis, the workers with schizophrenia took significantly longer sick leave compared to those with adjustment disorders. The details of this analysis are shown in Table 2.

**Table 2:** Predictors for duration of sick leave (univariate analysis)

Variables	Estimated mean days of sick leave	SE	95% CI		χ^2^	Degree of freedom	*p*
			Lower	Upper			
Socio-demographic							
Age at start of sick leave							
20–29	215.9	40.5	136.5	295.3	4.07	3	0.254
30–39	346.1	35.7	276.0	416.1			
40–49	331.8	41.8	250.0	413.7			
≧ 50	218.1	73.6	73.8	362.5			
Sex							
Male	318.6	26.9	265.9	371.3	0.33	1	0.564
Female	327.9	50.5	228.8	427.0			
Age at start of employment (years)							
< 25	331.8	36.4	260.5	403.0	1.16	2	0.561
25–29	339.0	40.3	259.9	418.0			
≧ 30	235.1	44.3	148.2	322.0			
Tenure (years)							
< 5	240.6	50.8	141.0	340.2	4.12	3	0.249
5–9	289.6	61.9	168.2	410.9			
10–19	322.4	35.2	253.4	391.5			
≧ 20	371.6	46.2	281.1	462.1			
Clinical							
Number of previous sick leave							
0	282.9	26.4	231.3	334.6	8.36	2	0.015
1–2	367.5	62.4	245.1	489.8			
≧ 3	444.8	72.7	302.4	587.3			
Diagnosis							
Major depressive disorder	330.2	33.3	265.0	395.4	13.96	5	0.016
Adjustment disorder	237.8	40.9	157.7	317.9			
Bipolar disorder	389.8	171.3	54.0	725.5			
Anxiety disorder	506.2	145.1	221.8	790.6			
Schizophrenia	586.9	130.0	332.0	841.7			
Others	328.2	69.8	191.4	465.0			
Work related							
Employee rank							
Manager	304.7	47.4	211.8	397.7	10.80	3	0.013
Senior staff	260.8	30.8	200.4	321.1			
Normal staff	378.0	45.6	288.7	467.4			
Assistant staff	663.3	123.4	421.4	905.1			

#### Multivariate analysis

We investigated multicollinearity among independent variables using Spearman’s rank correlation coefficient. The results showed a strong relationship between age and tenure (r = 0.81). Therefore, we conducted a Cox proportional hazard analysis for evaluating predictors of the duration of sick leave excluding tenure as a predictor variable. Table 3 shows the variables retained in the final Cox’s regression model with forced entry (sex and age) and stepwise forced selection procedure (all variables except sex and age). Three statistically significant predictors of the duration of sick leave were yielded by this process: age, number of previous sick leave, and employee rank, as discussed in more detail below.

**Table 3:** Predictors for duration of sick leave (multivariate analysis)

Predictor	*B*	*SE*	Wald	*Degree of freedom*	*p*	*Exp* (*B*)	Hazard ratio	
							Lower	Upper
Socio-demographic								
Age at start of sick leave^a^			15.66	3	0.001			
20–29						1.00	Reference	
30–39	− 1.05	0.31	11.44	1	0.001	0.35	0.19	0.64
40–49	− 1.19	0.37	10.61	1	0.001	0.30	0.15	0.62
≧ 50	− 0.28	0.50	0.32	1	0.57	0.75	0.28	2.00
Sex^a^								
Male						1.00	Reference	
Female	− 0.05	0.24	0.05	1	0.83	0.95	0.60	1.51
Clinical								
Number of previous sick leave^b^			6.39	2	0.04			
0						1.00	Reference	
1	− 0.24	0.25	0.91	1	0.34	0.79	0.48	1.29
2≦	− 0.71	0.29	6.16	1	0.01	0.49	0.28	0.86
Work related								
Employee rank^b^			20.12	3	< 0.001			
Manager						1.00	Reference	
Senior staff	0.44	0.28	2.49	1	0.12	1.55	0.90	2.66
Normal staff	− 0.44	0.35	1.53	1	0.22	0.65	0.32	1.29
Assistant staff	− 2.12	0.79	7.23	1	0.01	0.12	0.03	0.56

^a^Analysed using the forced entry method

^b^Analysed using the forward selection method (likelihood ratio)

In regard to age, the hazard ratios (95% CI) of 30–39 years, 40–49 years, and 50 years and older were 0.35 (0.19–0.64), 0.30 (0.15–0.62), and 0.75 (0.28–2.00), respectively, when 20–29 years was set to be the reference category.

With respect to the number of days of previous sick leave, when no history of previous sick leave was used as the reference, the hazard ratios of one previous episode, and two or more previous episodes were 0.79 (0.48–1.29) and 0.49 (0.28–0.86), respectively.

With respect to employee rank, the hazard ratios of senior staff, normal staff, and assistant staff were 1.55 (0.90–2.66), 0.65 (0.32–1.30), and 0.12 (0.03–0.56), respectively, when manager was set to be the reference category. Further details are shown in Table 3.

## Discussion

Considering that a large portion of the productivity loss caused by mental disorders occurs at the workplace, efforts to minimize the duration of sick leave are critical. As far as we know, this is the first study examining the predictors of duration of sick leave due to mental disorders among employees in Japan. Age, number of previous sick leave, and employee rank were found to be related to duration of sick leave.

### Socio-demographic factors

The duration of sick leave of participants in their 30 s and 40 s was significantly longer than that of those in their 20 s. A possible reason for this is the longer working hours of those in their 30 s and 40 s compared to other age groups in Japan [20]. Longer working hours may negatively affect the mental health condition of workers in their 30 s and 40 s, which could make their sick leave longer. A review of the prognostic factors of sick leave in Western countries showed that the duration of sick leave of workers in their 50 s was longer than in other age groups [12]. However, this study showed that the duration of sick leave in the 50 s age group was not significantly longer than in other age groups in Japan. One reason for this could be that the number of participants in their 50 s in this study was too small to detect statistical significance. An additional possibility could be that a selection bias known as the “healthy workers effect” might have occurred [21]. This takes place due to employees with mental disorders being more likely to leave the company. As a result, elder workers are likely to be mentally healthier than those of younger age.

### Clinical factors

The results in this study imply that the number of previous episodes of sick leave have a significant influence on sick leave duration. In preceding research, frequency of sick leave was related to work disability and job termination [22]. It is difficult for the persons with multiple sick leave to return to work, because of their work disability. It may follow longer sick leave and job termination in the end under the Japanese work system.

### Work-related factors

The results showed that employee rank was a predictor of duration of sick leave in Japan. One possible explanation of this result could be that the characteristics of the job roles in that company might have affected the results.

The absence duration of assistant staff was significantly longer than that of managers in this study. A possible reason for this could be that assistant staff might feel less pressure to return to work early because of the relatively lighter duties in their job. Therefore, it might be more difficult for them to be assigned a lighter workload on their return to work, as they already had light-duty jobs when they took sick leave, and this could therefore slow their return to work. Another reason for this result might be related to vulnerability of the workers. Considering that assistant staff suffer mental disorders under relatively light-duty, they are possibly more vulnerable to workplace stressors, and hence they needed to take more days off to recover compared with other employee ranks.

Senior staff showed the shortest duration of sick leave. As previously mentioned, senior staff are expected to perform at management-level as leaders of their team. In addition, they are also expected to work as non-executive employees. In Japan, this double role is known as “playing manager”. Many senior staff members have to perform this multiple role, and when they take sick leave, the impact of their absence on the workplace could be much more extensive than that of others. Thus, it could be possible that such work-related pressure might urge senior staff to return to work earlier.

This study has some limitations. Firstly, the participants in this study were derived from a single company and the sample size was relatively small. In order to be representative of all workers in Japan, future studies including a larger number of participants from a greater range of companies would be worthwhile. In addition, variables included in the analysis were mainly socio-demographic and clinical factors. There is a possibility that other factors such as severity of symptoms, work environment, and interpersonal relationships with colleagues could also be predictive factors of returning to work following sick leave [19, 23–26]. Therefore, future studies need to examine these factors. These limitations should be taken into consideration when interpreting the results of this study.

## Conclusion

Diagnosis, number of previous sick leave episodes, and employee rank are predictors of duration of sick leave due to mental disorders. This is the first study on predicting factors of the duration of sick leave due to mental disorders in Japan. When we consider more effective interventions to prevent protracted sick leave, those factors found by this study are quite critical. Future research is warranted on a larger scale of workers across a greater number of companies in Japan.

## Abbreviations

DSM-IV: the Diagnostic and Statistical Manual of Mental Disorders, Fourth Edition
JPY: Japanese Yen
USD: United States Dollars
SD: standard deviation
SE: standard error
